# Polymorphisms in Endothelin System Genes, Arsenic Levels and Obesity Risk

**DOI:** 10.1371/journal.pone.0118471

**Published:** 2015-03-23

**Authors:** Vanesa Martínez-Barquero, Griselda de Marco, Sergio Martínez-Hervas, Pilar Rentero, Inmaculada Galan-Chilet, Sebastian Blesa, David Morchon, Sonsoles Morcillo, Gemma Rojo, Juan Francisco Ascaso, José Tomás Real, Juan Carlos Martín-Escudero, Felipe Javier Chaves

**Affiliations:** 1 Department of Medicine, University of Valencia, Valencia, Spain; 2 Genotyping and Genetic Diagnosis Unit, Hospital Clínico Research Foundation (INCLIVA), Valencia, Spain; 3 CIBER of Diabetes and Associated Metabolic Diseases (CIBERDEM), Barcelona, Spain; 4 Service of Endocrinology and Nutrition, Hospital Regional Universitario, Málaga, Spain, Instituto de Biomedicina de Málaga (IBIMA), Málaga, Spain; 5 Service of Endocrinology and Nutrition, Hospital Clínico Universitario de Valencia, Valencia, Spain; 6 Internal Medicine, Rio Hortega Hospital, University of Valladolid, Valladolid, Spain; Tor Vergata University of Rome, ITALY

## Abstract

**Background/Objectives:**

Obesity has been linked to morbidity and mortality through increased risk for many chronic diseases. Endothelin (EDN) system has been related to endothelial function but it can be involved in lipid metabolism regulation: Receptor type A (EDNRA) activates lipolysis in adipocytes, the two endothelin receptors mediate arsenic-stimulated adipocyte dysfunction, and endothelin system can regulate adiposity by modulating adiponectin activity in different situations and, therefore, influence obesity development. The aim of the present study was to analyze if single nucleotide polymorphisms (SNPs) in the EDN system could be associated with human obesity.

**Subjects/Methods:**

We analyzed two samples of general-population-based studies from two different regions of Spain: the VALCAR Study, 468 subjects from the area of Valencia, and the Hortega Study, 1502 subjects from the area of Valladolid. Eighteen SNPs throughout five genes were analyzed using SNPlex.

**Results:**

We found associations for two polymorphisms of the EDNRB gene which codifies for EDN receptor type B. Genotypes AG and AA of the rs5351 were associated with a lower risk for obesity in the VALCAR sample (p=0.048, OR=0.63) and in the Hortega sample (p=0.001, OR=0.62). Moreover, in the rs3759475 polymorphism, genotypes CT and TT were also associated with lower risk for obesity in the Hortega sample (p=0.0037, OR=0.66) and in the VALCAR sample we found the same tendency (p=0.12, OR=0.70). Furthermore, upon studying the pooled population, we found a stronger association with obesity (p=0.0001, OR=0.61 and p=0.0008, OR=0.66 for rs5351 and rs3759475, respectively). Regarding plasma arsenic levels, we have found a positive association for the two SNPs studied with obesity risk in individuals with higher arsenic levels in plasma: rs5351 (p=0.0054, OR=0.51) and rs3759475 (p=0.009, OR=0.53)

**Conclusions:**

Our results support the hypothesis that polymorphisms of the EDNRB gene may influence the susceptibility to obesity and can interact with plasma arsenic levels.

## Introduction

Obesity is a state in which excess lipids accumulate in various body fat depots due to a chronic imbalance between energy intake and energy expenditure [1]. The prevalence of obesity is increasing. It is the most frequent metabolic disease, reaching epidemic proportions in industrialized countries [2]. Obesity significantly increases the risk of chronic diseases such as type 2 diabetes, cardiovascular disease, nonalcoholic fatty liver disease, colon cancer and obstructive sleep apnea [3].

Obesity is a proinflammatory condition in which both hypertrophied adipocytes and adipose tissue-resident immune cells (primarily lymphocytes and macrophages) contribute to the alteration of circulating levels of different molecules, including endothelins, affecting almost all tissues [4–7]. In obesity, endothelin system has an important role in endothelial dysfunction [8–9], and this may influence adipose tissue development [10].

The endothelin (EDN) system consists of three endothelin isoforms (EDN1, EDN2 and EDN3), and two receptors (endothelin type A and type B) linked to multiple signaling pathways [11]. Endothelins (EDN1, EDN2 and EDN3) are 21-amino acid peptides that exert their effects through their cognate receptors EDNRA and EDNRB.

EDNRA has high affinities for EDN1 and EDN2 and 100-fold lower affinity for EDN3, while EDNRB demonstrates equal affinities for all ligands [12,13]. Activation of EDNRA and EDNRB mediates many important functions, including vasoconstriction, cardiovascular remodeling, cell proliferation, cell differentiation, extracellular matrix production, and control of water and sodium secretion [13–15]. Although the pathophysiological role of EDNRA and EDNRB has not yet been completely elucidated, EDNRA is considered a bad receptor involved in pathogenesis and the development of various diseases as systemic and pulmonary hypertension, atherosclerosis, diabetes, and cardiac remodeling after myocardial ischemia [13, 15, 16]. EDNRB, however, has been classified as a good receptor since it functions as a “clearance receptor” preventing EDN1 from excessively stimulating EDNRA and enhancing endothelial production of nitric oxide (NO) and prostacyclin (PGI2) causing vasodilatation [16].

Endothelin can regulate adiponectin levels in different situations and therefore influence obesity development [10,17,18]. In addition, it has been described that EDNRA activates lipolysis in adipocytes [19], and the endothelin receptors mediate arsenic-stimulated adipocyte dysfunction [20]. Arsenic exposure has been linked to increased metabolic disease and alterations in lipid handling in adipose tissue [20, 21–25] and part of these alterations can be produced by an effect on EDN receptors inhibition [20, 26].

These data may indicate a role of the EDN system in obesity, and its genetic variation can modulate or be associated with obesity. Most genetic association studies of the EDN system have focused on other aspects, like the relationship with cardiovascular implications [27], asthma [28] and intracranial aneurysms [29, 30], but have not analyzed its relationship with obesity. Therefore, the aim of the present study was to analyze whether single nucleotide polymorphisms (SNPs) in the EDN system could be associated with human obesity.

## Material and Methods

### Population samples

We have analyzed two collections of general-population-based studies from two different regions of Spain: the VALCAR Study, with 468 subjects from the area of Valencia, and the Hortega Study, with 1502 subjects from the area of Valladolid. The sample tested included 1970 persons in total. The Hortega Study was approved by the Ethical Committee of Hospital Río Hortega, VALCAR Study and present genetic studies were approved by the Ethical Committee of Hospital Clínico Universitario de Valencia. All participants gave and signed their informed consent.

Demographic data (age and gender) along with anthropometric parameters were collected using standard procedures. The presence of obesity, hypertension (HTN) and type 2 diabetes was registered. BMI was calculated (kg/m^2^). Obesity, HTN and type 2 diabetes were defined using the World Health Organization (WHO) criteria (http://www.who.int). Briefly, obesity was diagnosed when a BMI value was ≥30 kg/m^2^. HTN was defined when systolic and diastolic blood pressure values were >140 or 90 mm Hg, respectively. The current WHO diagnostic criteria for diabetes were applied (http://www.who.int/diabetes/action_online/basics/en/index1.html): fasting plasma glucose ≥7.0mmol/l (126mg/dl) or 2 h plasma glucose ≥11.1mmol/l (200mg/dl). A previous diagnosis of type 2 diabetes or HTN and detection of the disease at the moment of sample collection was registered. Metabolic syndrome was defined by the National Cholesterol Education Program—Adult Treatment Panel (NCEP-ATP III) criteria [31] and by International Diabetes Federation IDF criteria [32].

Table 1 includes the general characteristics of the samples analyzed.

**Table 1:** General characteristics of the population.

Characteristic	Valcar	Hortega	Valcar + Hortega
No. Of subjects	468	1502	1970
Age (years)	46.4 ± 14.9	54.4 ± 19.3*	52.6 ± 18.7
BMI (kg/m2)	28 ± 4.7	26.4 ± 4.1*	27.7 ± 4.4
Waist circumference (cm)	92.6 ± 12.8	89.5 ± 13.0*	90.2 ± 13.0
Obesity (n, %)	114 (24.8)	356 (23.7) *	470 (23.9)
Abdominal obesity (n, %)	151 (32.8)	414 (27.6) *	565 (28.8)
Diabetes mellitus (n, %)	55 (11.9)	114 (7.6) *	169 (8.6)
Glucose (mg/dL)	99.7 ± 24.4	92.5 ± 20.4*	91.9 ± 29.9
Hypertension (n, %)	129 (26.7)	642 (42.7) *	771 (39.3)
Systolic blood pressure (mmHg)	126.5 ± 16.1	130.7 ± 21.6*	129.5 ± 20.5
Diastolic blood pressure (mmHg)	77.1 ± 9.9	79.1 ± 10.6*	78.2 ± 10.4
Total cholesterol (mg/dL)	222.7 ± 61.7	201.3 ± 38.1*	207.6 ± 46.2
HDL cholesterol (mg/ dL)	56.4 ± 15.1	51.7 ± 14.2*	53.9 ± 14.5
Triglycerides (mg/ dL)	123.4 ± 163.3	153.1 ± 113.8*	145.5 ± 127.4
Metabolic syndrome (n, %)			
IDF criteria	171 (36.5)	588 (39.1)*	759 (38.5)
ATP III criteria	148 (31.6)	480 (31.9)	628 (31.9)

Values are mean ± standard deviation. For qualitative variables, data are expressed as (n, %)

* *p-value < 0*.*0001*

The exclusion criteria were the same in both populations: the presence of serious concomitant diseases or disorders that could influence the collection of reliable information and any mental or social condition which might complicate or prevent the subject from participating in the study.

### SNP selection and genotyping

Venous blood samples were collected in tubes containing EDTA to obtain genomic DNA by the Chemagic system (Chemagen, Baesweiler, Germany). DNA was quantified and diluted to a final concentration of 100 ng ⁄μL.

EDN system SNPs for genotyping were selected based on the conjunction of several parameters by SYSNPs [33]: heterozygosity (>10% for the minor allele frequency) in a Caucasian population, position and spacing along the gene, and possible functional effect (http://www.ensembl.org/index.html). Eighteen SNPs from five genes were finally selected (Table 2). The SNPs were genotyped using an oligonucleotide ligation assay (SNPlex; Applied Biosystems, Foster City, CA, USA) following the manufacturer’s guidelines.

**Table 2:** Characteristics of selected Polymorphisms.

Chr	HGN	Gene name	Ensemble ID	SNP name	Chr position	Reference	% Genotype	HW	MAF	Allele Reference vs minor
6	EDN1	Endothelin 1	ENSG00000078401	rs1800541	12397205	NM_001955.4:c.-1644T>G	97.7	0.032	0.228	T>G
				rs3087459^¥^	12397625	NM_001955.4:c.-1224A>C	98.3	0.021	0.187	A>C
				rs2070699^¥^	12400758	NM_001955.4:c.233+30G>T	97.7	0.957	0.401	G>T
				rs5370^¥^	12404241	NM_001955.4:c.594G>T	98.5	0.170	0.206	G>T
1	EDN2	Endothelin 2	ENSG00000127129	rs11210262	41716731	NM_001956.3:c.*936G>C	97.0	0.476	0.072	G>C
				rs11572340^¥^	41723492	NM_001956.3:c.-633G>T	98.1	0.199	0.130	G>T
20	EDN3	Endothelin 3	ENSG00000124205	rs260741^¥^	57309550	NM_000114.2:c.52+236G>A	99.0	0.710	0.200	C>T
				rs11570257	57310195	NM_000114.2:c.365+23G>A	99.6	1.00	0.016	G>A
4	EDNRA	Endothelin receptor type A	ENSG00000151617	rs6842241^¥^	148620269	NM_001957.3:c.-1780C>A	97.3	0.123	0.247	C>A
				rs1801708	148621819	NM_001957.3:c.-230G>A	96.6	0.387	0.464	G>A
				rs5333	148680487	NM_001957.3:c.969T>C	99.5	0.256	0.326	T>C
				rs5334	148680523	NM_001957.3:c.1005G>A	98.3	0.440	0.326	G>A
				rs5335	148683290	NM_001957.3:c.*70G>C	98.9	0.927	0.499	G>C
				rs5342^¥^	148684221	NM_001957.3:c.*1001A>G	97.9	0.310	0.353	A>G
13	EDNRB	Endothelin receptor type B	ENSG00000136160	rs5352	77373231	NM_001201397.1:c.1184G>A	99.7	0.085	0.007	G>A
				rs5351	77373314	NM_001201397.1:c.1101A>G	99.4	0.788	0.451	G>A
				rs12716722^¥^	77390857	NM_001201397.1:c.220–97A>G	99.9	0.935	0.062	A>G
				rs3759475	77393106	NM_001201397.1:c.-1355T>C	99.7	0.604	0.500	C>T

HGN, The HUGO Gene Nomenclature.

^¥^:Tag-SNP by Hapmap in Caucasian patients. Ensemble ID: Ensemble human release 53. SNP name: dbSNP129 (NCBI´s build 36, version 3)

### Plasma Arsenic Level Assessment

Total arsenic was measured in the Hortega Study following a standardized protocol by inductively coupled-plasma mass spectrometry (ICPMS) with an Agilent 7500CEx ICPMS (Agilent Tecnologies, United States). The detection limit for arsenic plasma levels was 20.0 nmol/L.

### Statistical analysis

Samples with low genotyping results (<90%) were removed for all SNPs. All quantitative values are expressed as mean ± standard deviation or as (n, %) when describing qualitative variables. The ANOVA test was used to compare quantitative variables between groups and the chi-square test for categorical variables.

Allele and genotype frequencies were calculated for each SNP using SPSS and SNPSTATS (http://bioinfo.iconcologia.net/index.php). The Hardy—Weinberg equilibrium (HWE) was performed using a chi-square distribution with one degree of freedom using SNPSTAT software. The HWE was maintained for all the polymorphisms analyzed.

The association between polymorphisms and anthropometric parameters, BMI as a quantitative trait, and obesity as a qualitative trait, were examined first using a co-dominant inheritance model by SPSS and SNPSTAT, and afterward by the remaining models if two genotypes had similar means. The ANOVA procedure was used to compare mean differences for continuous variables among genotypes. The association of obesity with each polymorphism and haplotypes was sought using logistic regression models. Linkage disequilibrium measurements were calculated using the R2 statistic. The odds ratios (OR) were used to evaluate the risk for the presence of obesity for each polymorphism. Age and gender, as two potential confounders, were used as covariates. For multiple analysis corrections using Bonferroni correction, the p-value limit for significance for the study of 18 polymorphisms was 0.0027.

## Results

### Characteristics of the study population

The association between polymorphisms of the EDN system genes and the obesity traits was first analyzed in the VALCAR population. Positive results were analyzed in a second population, Hortega. Finally, the association was analyzed in all samples together.

The main characteristics of the two populations studied are shown in Table 1. The Valcar Study included 468 individuals (24.80% obese; 47.22% men) and the Hortega Study included 1502 subjects (23.70% obese; 49.47% men).

### Association of EDN system gene polymorphisms with obesity

Of all the polymorphisms, only two located in the EDNRB gene (rs5351 and rs3759475) were associated with obesity (Table 3). Polymorphisms with p<0.15 were selected and studied in Hortega Sample. Genotypes AG and AA of the rs5351 were associated with a lower risk for obesity in the Hortega sample (OR = 0.63; p = 0.048) and genotypes CT and TT of rs3759475 showed a tendency to lower risk for obesity (OR = 0.70; p = 0.12). These two polymorphisms showed similar results in VALCAR sample: genotypes AG and AA of rs5351 were associated with reduced risk of obesity (OR = 0.62; p = 0.001). Moreover, in the rs3759475 polymorphism, genotypes CT and TT were also associated with lower risk for obesity (OR = 0.66; p = 0.0037).

**Table 3:** Association between obesity and BMI and SNPs of EDNRB genes adjusted by age and sex in: A-Valcar Study / B-Hortega Study / C-Both.

EDNRB	dbSNP*/Reference	Genotype	N	BMI	% non obese	% obese	Obesity OR (95% CI)
**Gene**							
**A)**							
	rs5351						
	HWE: 0.7881	GG	154	27.43 (0.36)	105 (34.4%)	49 (46.2%)	1.00
	MAF: 0.451	AG-AA	257	27.45 (0.3)	200 (65.6%)	57 (53.8%)	0.63 (0.40–0.99)
	*P*-value			0.65			**0.048**
	rs3759475						
	HWE: 0.6045	CC	149	27.35 (0.37)	103 (33.8%)	46 (43.4%)	1.00
	MAF: 0.500	CT-TT	262	27.49 (0.3)	202 (66.2%)	60 (56.6%)	0.70 (0.44–1.10)
	*P*-value			0.41			0.12
**B)**							
	rs5351						
	HWE: 0.7881	GG	480	26.64 (0.21)	372 (31.9%)	108 (42.2%)	1.00
	MAF: 0.451	AG-AA	942	26.27 (0.13)	794 (68.1%)	148 (57.8%)	0.62 (0.47–0.83)
	*P*-value			0.08			**0.001**
	rs3759475						
	HWE: 0.6045	CC	487	26.63 (0.21)	379 (32.6%)	108 (41.7%)	1.00
	MAF: 0.500	CT-TT	935	26.32 (0.13)	784 (67.4%)	151 (58.3%)	0.66 (0.49–0.87)
	*P*-value			0.14			**0.0037**
**C)**							
	rs5351						
	HWE: 0.7881	GG	634	26.83 (0.18)	477 (32.4%)	157 (43.4%)	1.00
	MAF: 0.451	AG-AA	1199	26.52 (0.12)	994 (67.6%)	205 (56.6%)	0.61 (0.48–0.78)
	*P*-value			0.12			**1,00E-04**
	rs3759475						
	HWE: 0.6045	CC	636	26.8 (0.18)	482 (32.8%)	154 (42.2%)	1.00
	MAF: 0.500	CT-TT	1197	26.57 (0.12)	986 (67.2%)	211 (57.8%)	0.66 (0.52–0.84)
	*P*-value			0.29			**8,00E-04**

HWE, Hardy—Weinberg equilibrium; MAF, minor allele frequency; SNP, single-nucleotide polymorphism; OR, odds ratio. Values are mean ± standard error. Bold indicates significance.

*dbSNP 129.

Furthermore, upon studying the pooled population, we found a stronger association with obesity (p = 0.0001 and 0.0008 for rs5351 and rs3759475, respectively). We observed a slight tendency for association with lower BMI with the same genotypes.

### Association of EDN system gene polymorphisms with central obesity and other parameters

We have found a slight significant association with waist circumference of both polymorphisms when corrected by age and sex in Hortega sample but not in VALCAR study (see S1 Table). When we include BMI in the analysis the significance is lost.

Otherwise we have not found any association between EDNRB polymorphisms and other parameters as metabolic syndrome, HOMA-IR or glucose levels.

### Haplotype analysis

**Fig 1:**
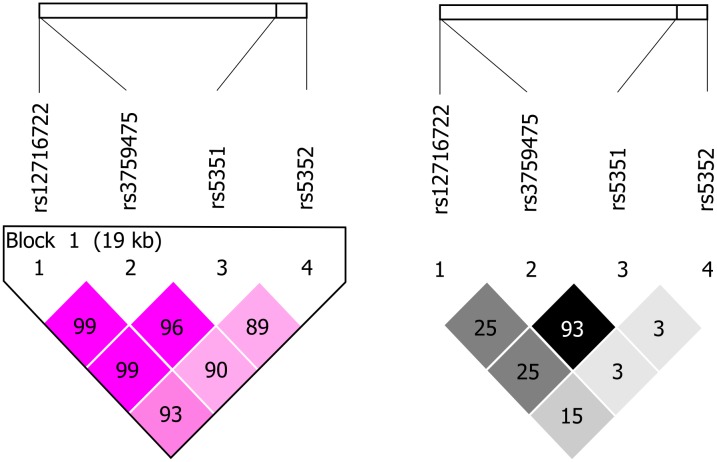
Linkage Disequilibrium (LD) plot of studied EDNRB SNPs. a) D´ b) R^2^.

Haplotype analysis was done with rs5351 and rs3759475 SNPs. One of the haplotypes including the two alleles associated with reduced risk of obesity, called number two in Table 4, was associated with lower risk for obesity. It was present in 40% of the individuals. These two polymorphisms have a high linkage disequilibrium (D´ = 0.967). Fig. 1 shows the linkage disequilibrium plots of the four studied EDNRB SNPs.

**Table 4:** Haplotype association analysis of rs5351 and rs3759475 of EDNRB gene with BMI and obesity risk adjusted by age and sex.

		Frequency					BMI	
No.	Haplotype	Non-obese	Obese	Freq	OR (95% CI)	P-value	Difference (95% CI)	P-value
1	GC	0.566	0.635	0.580	1.00	---	0.00	---
2	AT	0.418	0.347	0.403	0.73 (0.61–0.87)	**4,00E-04**	-0.22 (-0.49–0.05)	0.11
3	GT + AC	0.015	0.018	0.016	0.99 (0.52–1.88)	0.97	-0.32 (-1.39–0.74)	0.55

Single nucleotide polymorphisms used in the haplotype construction: rs5351, rs3759475.

### Association modulated by arsenic levels

We divided the Valladolid sample into tertiles by plasma arsenic levels, from individuals with lower arsenic levels (≤20 nmol/L), intermediate levels (>20 nmol/L and <50 nmol/L) and individuals with higher levels (≥50 nmol/L). We have observed a similar BMI media value in higher and medium arsenic serum levels tertiles (26.8 ± 4.1 kg/m^2^ and 26.5 ± 4.2 kg/m^2^ respectively). However lower arsenic serum levels tertil is associated with lower BMI media value (25.9 ± 4.3 kg/m^2^; p = 0.037; p = 0.002 and p = 0.003, when compared with second, third and with second and third tertiles together. In the analysis of the association of the rs5351 and rs3759475 polymorphisms with BMI and obesity, we found a trend for association of genotypes AG-AA of rs5351 and CT-TT of rs3759475 with lower BMI and a significant association with lower risk for obesity in individuals with higher arsenic levels, but not with those of medium or lower levels (see Table 5). In all cases, there is a similar trend. But in those with higher levels, the effect is stronger (the OR for rs5351 and for a population with higher levels is 0.50, and for the other groups it is 0.70; in rs3759475 the values are 0.50, 0.77 and 0.70 in the higher levels to the lower levels, respectively). Moreover, analysis by As tertiles do not show an association between ENDRB polymorphisms and waist.

**Table 5:** Relation with Arsenic serum levels.

1- Association with rs5351 (NM_001201397.1:c.1101A>G) of EDNRB gene.					
Genotype	N	BMI	% non obese	% obese	Obesity OR (95% CI)
**A) Individuals with high arsenic levels**					
GG	157	27.15 (0.36)	115 (30.4%)	42 (43.8%)	1.00
AG-AA	319	26.59 (0.22)	265 (69.6%)	54 (56.2%)	0.51 (0.32–0.82)
		0.08			**0.0054**
**B) Individuals with medium arsenic levels**					
GG	170	26.77 (0.35)	133 (34.2%)	37 (43.0%)	1.00
AG-AA	305	26.32 (0.23)	256 (65.8%)	49 (57.0%)	0.70 (0.43–1.13)
		0.29			0.15
**C) Individuals with low arsenic levels**					
GG	152	25.98 (0.37)	123 (31.1%)	29 (38.7%)	1.00
AG-AA	318	25.92 (0.23)	272 (68.9%)	46 (61.3%)	0.70 (0.41–1.20)
		0.71			0.20

**2- Association with rs3759475 (NM_001201397.1:c.-1355T>C) of EDNRB gene.**					
**Genotype**	**N**	**BMI**	**% non obese**	**% obese**	**Obesity OR (95% CI)**
**A) Individuals with high arsenic levels**					
CC	164	27.21 (0.34)	120 (32.0%)	44 (44.9%)	1.00
CT-TT	310	26.65 (0.22)	256 (68.0%)	54 (55.1%)	0.53 (0.33–0.85)
		0.093			**0.009**
**B) Individuals with medium arsenic levels**					
CC	167	26.73 (0.35)	132 (33.8%)	35 (40.2%)	1.00
CT-TT	311	26.37 (0.22)	259 (66.2%)	52 (59.8%)	0.77 (0.47–1.25)
		0.39			0.30
**C) Individuals with low arsenic levels**					
CC	155	25.90 (0.37)	126 (32.0%)	29 (38.7%)	1.00
CT-TT	314	25.96 (0.23)	268 (68.0%)	46 (61.3%)	0.70 (0.41–1.21)
		0.79			0.20

Association between obesity and BMI and SNPs of EDNRB genes adjusted by age and sex. OR, odds ratio. Values are mean ± standard error. Bold indicates significance.

### Haplotype analysis in relation to arsenic levels

Haplotype analysis in the tertiles based on arsenic levels were performed for the rs5351 and rs3759475 SNPs (Table 6). Haplotype 2 remains associated with lower obesity risk only in the higher arsenic level group.

**Table 6:** Haplotype association analysis of rs5351 and rs3759475 of EDNRB gene with BMI and obesity risk adjusted by age and sex in relation with Arsenic serum levels.

A) Individuals with higher Arsenic serum levels.								
		Frequency					BMI	
No.	Haplotype	Non-obese	Obese	Freq	OR (95% CI)	P-value	Difference (95% CI)	P-value
1	GC	0.549	0.678	0.576	1.00	---	0.00	---
2	AT	0.430	0.306	0.405	0.53 (0.37–0.76)	**6,00E-04**	-0.49 (-1.01–0.02)	0.06
3	AC	0.012	0.010	0.012	0.66 (0.13–3.28)	0.62	0.02 (-2.35–2.39)	0.99
4	GT	0.008	0.005	0.007	0.78 (0.09–6.72)	0.82	-0.32 (-3.24–2.6)	0.83

**B) Individuals with medium Arsenic serum levels.**								
		Frequency					BMI	
No.	Haplotype	Non-obese	Obese	Freq	OR (95% CI)	P-value	Difference (95% CI)	P-value
1	GC	0.580	0.620	0.587	1.00	---	0.00	---
2	AT	0.402	0.356	0.393	0.84 (0.59–1.20)	0.34	-0.27 (-0.79–0.25)	0.31
3	AC	0.008	0.006	0.007	0.69 (0.08–6.05)	0.74	-2.05 (-5.01–0.91)	0.18
4	GT	0.010	0.018	0.012	2.00 (0.49–8.11)	0.33	0.27 (-2.1–2.64)	0.82

**C) Individuals with lower Arsenic serum levels.**								
		Frequency					BMI	
No.	Haplotype	Non-obese	Obese	Freq	OR (95% CI)	P-value	Difference (95% CI)	P-value
1	GC	0.551	0.599	0.558	1.00	---	0.00	---
2	AT	0.435	0.395	0.429	0.82 (0.56–1.19)	0.29	-0.13 (-0.62–0.36)	0.61
3+4	AC+GT	0.014	0.007	0.013	0.30 (0.04–2.52)	0.27	-1.96 (-4.19–0.27)	0.08

Single nucleotide polymorphisms used in the haplotype construction: rs5351, rs3759475.

## Discussion

We have genotyped eighteen polymorphisms located in the five genes of the EDN system and we have analyzed their association with BMI and obesity. We have found an association for two polymorphisms of the EDNRB gene with obesity. Genotypes AG and AA of the rs5351 were associated with a lower risk for obesity in both samples and overall when we analyzed the pooled sample. Moreover, in the rs3759475 polymorphism, genotypes CT and TT showed a trend for lower risk in the VALCAR sample and were associated with a lower risk for obesity in the Hortega population. The effect increased when the samples were analyzed together. Regarding the BMI data, we have found a trend congruent with these results although the values do not reach statistical significance. Otherwise our data show the absence of association of rs5351 and rs3759475 with waist or abdominal obesity although we have found a slight association lost when BMI is included as a covariable in the analysis.

We have analyzed if EDNRB polymorphisms were associated with glucose levels and metabolic syndrome (defined by ATP III and IDF criteria) and we have not found any association of rs5351 and rs3759475 with these parameters. These data could indicate the possible importance of these polymorphisms in obesity risk but not in metabolic complications, although specific studies focused in these parameters should be done to clarify this point.

It is remarkable that although the Hortega and VALCAR populations had different characteristics, the findings were replicated and the analysis of whole sample showed a stronger association. As noted in Table 4, a haplotype associated with a lower risk for obesity and which is frequent in the population, is congruent with the data obtained in individual polymorphism analysis (haplotype AT for rs5351 and rs3759475).

The functional significance of these two SNPs is not clear. SNP rs5351 is located in exon 6, it is a synonymous variation in this gene, and it is present in the transcript of EDNRB antisense RNA 1 (EDNRB-AS1) without a known function. And rs3759475 is an intronic variation. Nevertheless, it should be noted that both have a MAF >0.40, and that they have a high linkage disequilibrium (D´ = 0.967). There are no studies including the associated SNPs found in this work, except for rs5351, which has been associated with atherosclerosis [27]. Nevertheless, there are studies that identify EDNRB (and rs5351 in particular), as a gene with significant allelic expression difference. Differential allelic expression can be associated with differences due to genetic variations or epigenetic mechanisms involved in gene regulation [34]. In addition, these polymorphisms can be in linkage disequilibrium with functional variations of this gene.

Regarding plasma arsenic levels, we have found an association between increased levels and higher BMI levels as previously described [35]. We have observed a positive association for the two SNPs studied (rs5351 and rs3759475) with obesity risk in individuals with higher arsenic levels. The haplotype analysis showed that the same haplotype found in the pooled population was associated with a lower risk for obesity in individuals with higher arsenic levels, while there was no association in those with medium or lower arsenic levels (Table 6). These data show that arsenic can modulate the effect of the genetic polymorphisms of the EDNRB gene in modulating obesity.

This association has not been previously found by GWAS studies. These differences may be the result of different environmental factors in the populations included in the different studies, like differences in nutrition or in arsenic levels derived from it. Another explanation may come from the specific situation of a Mediterranean population. Most of the time it is underrepresented in GWAS studies, and many times there is no Spanish sample in these studies [36–38]. Nevertheless, there are other studies in Spanish population of obesity-related genes described in different populations but FTO was the only locus that was clearly associated with BMI while the other 22 analyzed were not associated [39]. Considering the high heritability of obesity, new variants remain to be discovered and, overall, in Spanish population where previously described polymorphisms seem not to have the effect previously described.

Most of the work done up to now in relation to the endothelin system and obesity has been done in relation to increased levels of endothelin in obesity and their possible effects on vascular tone and endothelial disfunction [40]. Some works indicate that endothelin is involved in obesity through different mechanisms such as the regulation of blood flow to adipose tissue [10]. In addition, endothelin system can regulate some hormones involved in adipogenesis and metabolic processes that can be related to obesity. Thus, endothelin has been identified as a regulator of adiponectin levels in obese children and as a regulator of adiponectin gene expression and secretion [17,18]. It also regulates glucose update, glycolysis and lipolysis genes in rats [19, 41–44]. Most of these effects seem to be mediated by EDNRA, while there is little information about a possible role of EDNRB. Nevertheless, EDNRB has been involved in peripheral microvascular function, cancer development and in the development of different hereditary diseases like Waardenburg syndrome or Hirschsprung disease and, therefore, EDNRB is also involved in the alteration of enteric neurons [45–48]. Moreover, this protein is related to the development and growth of different tumors [3,4]. All these facts can link this gene with obesity by different pathways (cell differentiation, growth, neuronal processes, etc.). In addition, it has been described that inhibition of EDNRB protects lipid droplets against the effect of arsenic [20], which can be associated with the development of obesity. Thus, our results indicate that elevated arsenic levels modulate the EDNRB association with obesity.

Otherwise EDN system can modulate the toxic effect of arsenic. EDNRA and EDNRB signaling is often cooperative with the formation of heterodimeric signaling complexes suggesting that arsenic may be acting through a complex of EDNR [20]. Therefore, polymorphisms affecting the activity of EDN receptors by modification of their sequence, their expression levels or their regulation can be involved in different responses to arsenic exposure. The effect of arsenic or the inhibition of these receptors can modulate the perilipin 1 (PLIN1) expression and lipolysis and, therefore, obesity risk and its consequences. All previous data may indicate that activity of EDN system can modulate the toxic effect of Arsenic.

### Limitations of our study

The number of individuals included in the present study is limited, but the statistical power is sufficient for the polymorphisms analyzed. The number of polymorphisms analyzed is reduced, nevertheless we do not expect to study total genetic variability in these genes because many other polymorphisms may be analyzed in these genes and studies with a higher number of polymorphisms would be required. We have only measured arsenic levels in one of the samples, although it is the larger of the two. Finally, the functional meaning of these two SNPs with a significant association is not clear, and other studies should be performed in order to identify whether these polymorphisms or others in linkage disequilibrium have a functional effect and how they can be involved in modulating obesity risk.

In conclusion, our study carried out in two Spanish populations from different regions and noticeably distinct characteristics supports the hypothesis that polymorphisms of the EDNRB gene may influence the susceptibility to obesity. In addition, blood arsenic levels can modulate the influence of these polymorphisms on obesity risk.

Despite this evidence, we should be cautious in the interpretation of the data. The possible role of SNPs of the EDNRB gene in obesity phenotypes should be confirmed by other studies, including analyses among different populations, larger sample sizes, etc.

## Supporting Information

S1 Table Association between central obesity and waist and SNPs of EDNRB genes adjusted by age and sex in: A-Valcar Study / B-Hortega Study / C-Both. (DOC)
