# Supplementary material for: Polymorphisms in Endothelin System Genes, Arsenic Levels and Obesity Risk
Source: PLoS One. 2015 Mar 23;10(3):e0118471. doi: 10.1371/journal.pone.0118471 (PMC4370725; doi:10.1371/journal.pone.0118471)
Supplement: S1 Table — (DOC) [file pone.0118471.s001.doc]

| **S1 Table.** Association between central obesity and waist and SNPs of EDNRB genes adjusted by age and sex in: A-Valcar Study / B-Hortega Study / C-Both. | | | | | | | |
| --- | --- | --- | --- | --- | --- | --- | --- |
| **EDNRB** | **dbSNP*/Reference** | **Genotype** | **N** | **Waist** | **% non obese** | **% obese** | **Central Obesity OR (95% CI)** |
| **Gene** |  |  |  |  |  |  |  |
| **A)** |  |  |  |  |  |  |  |
|  | rs5351 (NM_001201397.1:c.1101A>G) |  |  |  |  |  |  |
|  | HWE: 0.7881 | GG | 145 | 92.34 (1.05) | 47 (38.5%) | 105 (36.8%) | 1.00 |
|  | MAF: 0.451 | AG-AA | 245 | 92.38 (0.83) | 75 (61.5%) | 180 (63.2%) | 1.19 (0.73-1.93) |
|  | *P*-value |  |  | 0.73 |  |  | 0.48 |
|  |  |  |  |  |  |  |  |
|  | rs3759475 (NM_001201397.1:c.-1355T>C) |  |  |  |  |  |  |
|  | HWE: 0.6045 | CC | 140 | 92.02 (1.06) | 47 (38.5%) | 100 (35.1%) | 1.00 |
|  | MAF: 0.500 | CT-TT | 250 | 92.56 (0.82) | 75 (61.5%) | 185 (64.9%) | 1.33 (0.81-2.16) |
|  | *P*-value |  |  | 0.40 |  |  | 0.26 |
|  |  |  |  |  |  |  |  |
| **B)** |  |  |  |  |  |  |  |
|  | rs5351 (NM_001201397.1:c.1101A>G) |  |  |  |  |  |  |
|  | HWE: 0.7881 | GG | 476 | 90.5 (0.62) | 332 (32.2%) | 145 (36.4%) | 1.00 |
|  | MAF: 0.451 | AG-AA | 951 | 89.01 (0.41) | 698 (67.8%) | 253 (63.6%) | 0.77 (0.59-1.01 |
|  | *P*-value |  |  | **0.011** |  |  | 0.061 |
|  |  |  |  |  |  |  |  |
|  | rs3759475 (NM_001201397.1:c.-1355T>C) |  |  |  |  |  |  |
|  | HWE: 0.6045 | CC | 486 | 90.32 (0.62) | 340 (33.1%) | 147 (36.5%) | 1.00 |
|  | MAF: 0.500 | CT-TT | 943 | 89.26 (0.41) | 687 (66.9%) | 256 (63.5%) | 0.81 (0.62-1.06 |
|  | *P*-value |  |  | **0.049** |  |  | **0.13** |
|  |  |  |  |  |  |  |  |
| **C)** |  |  |  |  |  |  |  |
|  | rs5351 (NM_001201397.1:c.1101A>G) |  |  |  |  |  |  |
|  | HWE: 0.7881 | GG | 621 | 90.89 (0.54) | 381 (32.9%) | 250 (36.6%) | 1.00 |
|  | MAF: 0.451 | AG-AA | 1196 | 89.7 (0.37) | 773 (67.1%) | 433 (63.4%) | 0.83 (0.67-1.02) |
|  | *P*-value |  |  | **0.028** |  |  | 0.075 |
|  |  |  |  |  |  |  |  |
|  | rs3759475 (NM_001201397.1:c.-1355T>C) |  |  |  |  |  |  |
|  | HWE: 0.6045 | CC | 624 | 90.67 (0.54) | 387 (33.7%) | 247 (35.9%) | 1.00 |
|  | MAF: 0.500 | CT-TT | 1193 | 89.95 (0.37) | 762 (66.3%) | 441 (64.1%) | 0.90 (0.73-1.11) |
|  | *P*-value |  |  | 0.19 |  |  | 0.31 |
|  |  |  |  |  |  |  |  |
| HWE, Hardy–Weinberg equilibrium; MAF, minor allele frequency; SNP, single-nucleotide polymorphism; OR, odds ratio. | | | |  |  |  |  |
| Values are mean ± standard error. | |  |  |  |  |  |  |
| Bold indicates significance. | |  |  |  |  |  |  |
| *dbSNP 126. | |  |  |  |  |  |  |
